# Common Genetic Risk for Melanoma Encourages Preventive Behavior Change

**DOI:** 10.3390/jpm5010036

**Published:** 2015-02-17

**Authors:** Lori Diseati, Laura B. Scheinfeldt, Rachel S. Kasper, Ruixue Zhaoyang, Neda Gharani, Tara J. Schmidlen, Erynn S. Gordon, Cecili K. Sessions, Susan K. Delaney, Joseph P. Jarvis, Norman Gerry, Michael Christman

**Affiliations:** 1 United States Air Force Medical Services, 7700 Arlington Boulevard, Falls Church, VA 22042, USA; E-Mails: lori.diseati@us.af.mil (L.D.); cecili.sessions@us.af.mil (C.K.S.); 2 Coriell Institute for Medical Research, 403 Haddon Ave, Camden, NJ 08103, USA; E-Mails: lscheinfeldt@coriell.org (L.B.S.); rskasper@comcast.net (R.S.K.); rzhaoyang@coriell.org (R.Z.); gharani.consulting@gmail.com (N.G.); tschmidlen@coriell.org (T.J.S.); egordon@23andme.com (E.S.G.); sdelaney@coriell.org (S.K.D.); jjarvis@coriell.org (J.P.J.); ngerry@coriell.org (N.G); 3 23andMe, Inc., 1390 Shorebird Way, Mountain View, CA 94043, USA

**Keywords:** melanoma, prevention, genetic

## Abstract

There is currently great interest in using genetic risk estimates for common disease in personalized healthcare. Here we assess melanoma risk-related preventive behavioral change in the context of the Coriell Personalized Medicine Collaborative (CPMC). As part of on-going reporting activities within the project, participants received a personalized risk assessment including information related to their own self-reported family history of melanoma and a genetic risk variant showing a moderate effect size (1.7, 3.0 respectively for heterozygous and homozygous individuals). Participants who opted to view their report were sent an optional outcome survey assessing risk perception and behavioral change in the months that followed. Participants that report family history risk, genetic risk, or both risk factors for melanoma were significantly more likely to increase skin cancer preventive behaviors when compared to participants with neither risk factor (ORs = 2.04, 2.79, 4.06 and *p*-values = 0.02, 2.86 × 10^−5^, 4.67 × 10^−5^, respectively), and we found the relationship between risk information and behavior to be partially mediated by anxiety. Genomic risk assessments appear to encourage positive behavioral change in a manner that is complementary to family history risk information and therefore may represent a useful addition to standard of care for melanoma prevention.

## 1. Introduction

Personalized medicine promises customized individual healthcare that goes beyond the genetic screening already commonplace in obstetrics, oncology, and pharmacology [1]. However, it remains unclear how probabilistic rather than diagnostic or functional genetic information can contribute to improving health outcomes [2], or if such information produces adverse psychological effects such as anxiety and/or wasteful over-utilization of healthcare services [3]. As the pace of genomic research accelerates, two primary challenges will be to identify research results with potential clinical utility and successfully translate them into meaningful and constructive additions to the current standard of care.

One way to incorporate genetic information into the clinical setting is as a motivational tool encouraging preventive clinical testing and patient behavioral change. Indeed, a robust understanding of the impact of genetic testing on preventive health behaviors for individuals with increased risk of common complex diseases such as cardiovascular disease, diabetes mellitus, and cancer would represent a great public health advance [4].

In the case of melanoma, a form of skin cancer that affects melanocytes, behavioral modifications such as skin self-examination, the use of sunscreen and protective clothing, and avoidance of UV light exposure represent relatively simple and accessible ways to reduce the overall risk of developing the disease [5]. In 2013, there were almost 77,000 new cases and 10,000 deaths due to melanoma in the United States with a rising incidence and prevalence [5]. However, by self-report, only one-third of the US population regularly applies sunscreen; 41% regularly wear fully sun-protective clothing; and only 32% regularly choose sun avoidance [5].

Hereditary melanoma has been associated with mutations in the p16 (CDKN2A) gene, and multiple studies have evaluated the impact of p16 testing on preventive behaviors related to melanoma. Aspinwall *et al.* [6] found that individuals with CDKN2A/p16 mutations were more likely to increase skin self-exams after receiving their genetic test results (*p* < 0.002) in a prospective study of 59 participants. Similarly, Kasparian *et al.* [7] report a significant increase in clinical skin exams in individuals with a positive CDKN2A/p16 genetic result (*p* = 0.02) in a prospective study of 119 participants. More recently, two studies found that after p16 testing intent to increase sun protective behaviors [8], short term sun protective behaviors [8], and long term sun protective behaviors [9] improved. 

It is unclear, however, to what degree screening for common genetic risk factors for melanoma would increase preventive behaviors among individuals who are not at increased risk for hereditary melanoma. In the current study we address this question in a large prospective study (*n* = 718) of healthy participants using a common genetic variant (>2% globally) [10], rs910873, located in the PIGU gene that is associated with increased risk of melanoma (1.7× for heterozygotes and 3× for homozygotes carrying the T allele) in the general population [11]. We have analyzed self-reported data from the Coriell Personalized Medicine Collaborative (CPMC) and demonstrate that common genetic risk factors for melanoma play a significant role in motivating preventive behaviors that mitigate the risk of developing melanoma (*i.e.*, reduced sun exposure, increased use of sunscreen, protective clothing, and skin self-examinations). Furthermore, we have found that the association between increased risk for melanoma and increased preventive behaviors is partially mediated by anxiety.

## 2. Results and Discussion

### 2.1. Results

As part of the ongoing CPMC, which is a prospective research study focused on the impact that disease risk assessment has on health outcomes [12,13], participants are given personalized melanoma risk reports that include genetic and family history risk factors for melanoma and subsequently (at least 3 months after viewing the risk report) asked to complete optional outcome surveys that capture what they did with the information. Participants in the current study were recruited through one of three cohorts: CPMC community cohort, The Ohio State University (OSU) community cohort, or the United States Air Force Medical Service cohort (Table 1).

**Table 1:** Participant demographics.

*n*	718
age in years, mean (range)	52.86 (21–91)
male, *n* (%)	245 (34.12)
female, *n* (%)	473 (65.88)
Air Force Medical Service, *n* (%)	118 (16.43)
Coriell Personalized Medicine Collaborative community, *n* (%)	498 (69.36)
The Ohio State University community, *n* (%)	102 (14.21)

As described in more detail elsewhere [12,13], family history risk is collected through self-reported questionnaires, and genetic risk is evaluated through extensive literature review and by the Coriell Informed Cohort Oversight Board (ICOB). For the melanoma risk report, genetic risk evidence presented in Brown *et al.* [11] was approved by the ICOB, genotyped at Coriell’s CLIA-certified genotyping laboratory, and used in the CPMC risk report. Risk reports are available through CPMC’s online portal (an example of a melanoma risk report can be found at the following web address: https://cpmc.coriell.org/v/Report/Demo/Melanoma/DemoNat), and participants choose which, if any, risk reports they want to view [14].

We were interested in examining whether participant understanding of their reported risk factors for melanoma was associated with behavior changes that mitigate melanoma risk. To address this question, we first asked if there was any significant difference among participants reporting a particular risk category with regard to preventive behavior change. We defined preventive behavior change as “no” or “yes”, where “yes” indicates the increase of one or more of the following protective behaviors: decreased sun exposure or increased sunscreen, protective clothing or self-skin examinations. We defined risk category as follows: no risk factors, family history risk only, genetic risk (one or two copies of the genetic risk allele) only, or both family history and genetic risk factors. We used ANOVA to test for any difference among the four risk categories with respect to preventive behavior, and indeed found a significant difference among groups (F-value = 36.93; *p* < 1.99 × 10^−9^).

**Figure 1:**
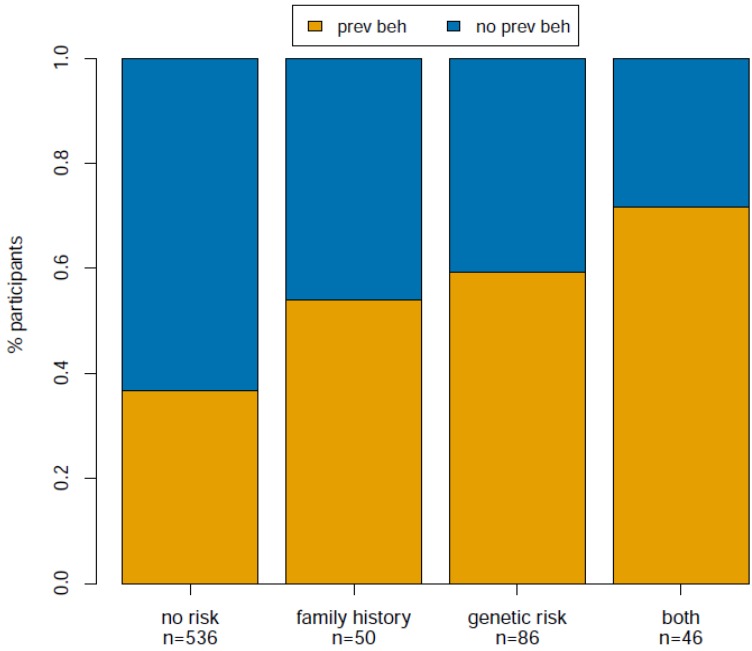
Proportion of participants that adopted preventive behaviors after viewing their melanoma risk reports.

To further explore the relationship between risk category and preventive behavior we used binomial logistic regression to test for association between preventive behavior and risk category after correcting for age, gender and cohort (see Table 2). We found that all three increased risk categories (family history, genetic risk, both) are significantly more likely to result in preventive behaviors than the no risk category (*p* = 0.02, 2.86 × 10^−5^, 4.67 × 10^−5^, respectively), and the magnitude of the effect increases such that family history is the lowest (OR = 2.04), genetic risk is intermediate (OR = 2.79), and both is the highest (OR = 4.06). This result is also illustrated in Figure 1, which displays the raw proportion of individuals in each risk category that increased preventive behaviors after viewing their CPMC melanoma risk report. Comparisons among the three increased risk groups and comparison between participants with one and two copies of the genetic risk variant were not statistically significant (*p* > 0.40). In addition, we found similar results when analyzing skin exams and sun protection as separate outcome variables (Table A1 and Table A2).

**Table 2:** Logistic regression modeling results for preventive behavior change and melanoma risk.

	OR	eta	SE	z Value	*p* Value
(Intercept)	0.20	−1.61	0.36	−4.43	9.25 e-06
family history (*vs.* no risk)	2.04	0.71	0.30	2.36	0.02
genetic (*vs.* no risk)	2.79	1.03	0.25	4.18	2.86 e-05
both (*vs.* no risk)	4.06	1.40	0.34	4.07	4.67 e-05

Given the significant association between risk category and preventive behaviors after correcting for age, gender, and cohort, we were interested in further exploring this relationship. We next incorporated reported anxiety levels after viewing the melanoma risk report. We used binomial logistic regression to incorporate anxiety into our model and found that after correcting for age, gender, and cohort, the significance level (*p*-value = 9.64 × 10^−9^) and the magnitude of effect (eta = 0.91) were more significant and stronger, respectively for anxiety than for any of the increased risk categories (see Table 3). No interaction terms between risk category and demographic covariates were significant (*p* > 0.20).

**Table 3:** Logistic regression modeling results for preventive behaviors, anxiety and melanoma risk.

Column1	OR	eta	SE	z Value	*p* Value
(Intercept)	0.07	−2.62	0.41	−6.34	2.36 e-10
anxiety	NA	0.91	0.16	5.74	9.64 e-09
family history (*vs.* no risk)	1.71	0.53	0.31	1.71	0.09
genetic risk (*vs.* no risk)	1.93	0.66	0.26	2.51	0.01
both (*vs.* no risk)	2.35	0.86	0.37	2.33	0.02

**Figure A1:**
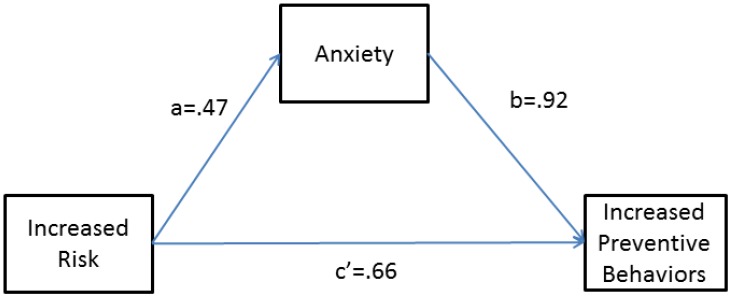
Anxiety mediation model. The relationship between anxiety, melanoma risk and preventive behaviors is visualized below.

In order to better understand the relationship among preventive behavior, risk category, and participant anxiety levels (1–5 Likert-type scale) after viewing the melanoma risk report, we tested a simple mediation model [15] after correcting for demographic covariates (age, gender, and recruitment cohort). In particular, we hypothesized that risk factors for melanoma may have influenced preventive behavior change through feelings of anxiety such that increased risk (genetic or family history) predicts increased anxiety, which in turn predicts the chance of adopting or increasing preventive behaviors. The proposed mediation model is diagrammed in Figure A1 , and as shown, the model accounts for the impact of risk factors on anxiety, the impact of anxiety on preventive behaviors, and the impact of risk on preventive behaviors directly. We found that the effect of any increased risk category relative to no risk category on preventive behavior change is partially mediated by increased anxiety levels after viewing the melanoma risk report. More specifically, we found that risk factor significantly predicts increased anxiety (beta = 0.47, OR = 1.6, *p*-value < 1.00 × 10^−4^, path “a” in Figure A1), which in turn significantly predicts the chance that a participant has adopted a preventive behavior after viewing the melanoma risk report (beta = 0.92, OR = 2.51, *p*-value < 1.00 × 10^−4^, path “b” in Figure A1). Additionally, the direct effect of risk factor on preventive behavior, which quantifies the effect of risk factor on preventive behavior that is independent of anxiety, was also significant (beta = 0.66, OR = 1.93, *p*-value = 7.00 × 10^−4^, path “c'’’ in Figure A1 ). The indirect effect of risk factor on preventive behavior (a*b) is 0.44, and the total effect of risk category on preventive behavior is 1.03 (*p*-value < 1.00 × 10^−4^). Hayes [15] notes that in models of dichotomous traits, the total effect is not always equal to the sum of the direct and indirect effects. Overall, the mediation analysis indicates that anxiety after viewing the melanoma risk report partially explains the association between risk factor and preventive behaviors; however, even after taking this relationship into account, risk factor is still significantly impacting preventive behavior.

Finally, we examined two additional questions included in the outcome survey related to preventive behavior motivations. More specifically, participants were asked what motivated them to make a particular reported behavior change by choosing all applicable responses from the following: “my CPMC genetic variant result for melanoma”, “my CPMC family history result for melanoma”, “I had symptoms of melanoma”, “my CPMC results for other conditions”, “my health care provider’s recommendations”, “I have/had another type of skin cancer (basal cell, squamous cell, *etc*.)”, or “other”. We compared the number of participants who reported only “genetic variant” (*n* = 65 out of 308 participants that increased preventive behaviors) with the number of participants who reported only “family history” (*n* = 32 out of 308 participants that increased preventive behaviors), and found significantly more participants report being motivated by their genetic result (*X*^2^ = 12.53, *p*-value = 4.01 × 10^−4^). This comparison is still significant when restricting to participants that reported genetic risk (65/86) or family history risk (25/50), respectively (*X*^2^ = 8.14, *p*-value=4.33 × 10^−3^). In addition, 23 out of 46 participants that reported both risk factors also reported being motivated by both genetic and family history risk. This proportion was significantly fewer than those who only reported being motivated by genetic risk (*X*^2^ = 7.71, *p*-value = 5.49 × 10^−3^) and not significantly different from those who only reported being motivated by family history (*X*^2^ = 0.00, *p*-value = 1.00).

### 2.2. Discussion

To our knowledge, this is the largest study (*n* = 718) to establish an association between reported genetic risk of melanoma and preventive behaviors, and the only study to focus on common genetic variants rather than familial cases of melanoma. We found that any increased risk for melanoma (increased genetic risk, increased family history risk, or increased genetic and family history risk) is significantly associated with increased preventive behaviors, and this association is partially mediated by anxiety. Moreover, significantly more participants that did increase preventive behaviors reported that they were motivated by their genetic risk rather than by family history risk for melanoma.

The US Preventive Services Task Force and others (e.g., [16]) already emphasize the importance of family history in assessing disease risk in the primary care setting, and our results suggest that genetic testing for melanoma risk will complement the current standard of care in motivating preventive behaviors. Furthermore, there are situations in which family history is either not known (e.g., adopted individuals), incompletely known, or incorrectly known that may be particularly suitable for supplementary genetic testing.

It is worth noting that in many respects melanoma represents a “best case scenario” for incorporating genetic risk for complex disease into preventive clinical care. Indeed, studies of behavior change related to other conditions have not been as encouraging [17]. First, there are several lifestyle changes that mitigate the risk of melanoma that are accessible and affordable to everyone (e.g., avoiding direct sunlight and wearing protective clothing outside). Second, skin exams are non-invasive clinical screening tools for melanoma. Third, early detection of melanoma has profound impact on prognosis [18]. Therefore, genetic risk assessment may not be appropriate for other conditions where risk mitigation is undefined or inaccessible, clinical screening is invasive and/or poses risk to patients, or prognosis is not improved by early detection. Moreover, in the CPMC melanoma risk assessment, the reported risk factors are not deterministic, but rather contribute to increased risk for the disease relative to individuals without the risk factors. The maximum genetic and family history relative risk a participant can have is 3 and 2.2, respectively. We acknowledge the possibility that participants may be “over-interpreting” their risk; however, we did not evaluate this in the current study. In cases where interventions based on genetic risk factors pose additional risks to patients, appropriate understanding of genetic risk factors will be critical.

To explore any potentially negative impact to participants’ state of mind, we evaluated levels of anxiety after viewing the melanoma risk report. We found that only 2 (0.3%) participants reported high, and 29 (4%) participants reported moderate levels of anxiety. The vast majority (74%) of participants that did report moderate or high levels of anxiety reported having at least one risk factor for melanoma, which suggests that genomic testing may contribute to a heightened level of anxiety which may cause individuals to take actions to further address and investigate the implications of disease.

One hundred participants (14%) reported sharing their CPMC risk report for melanoma with a health care provider. Health care providers for 17 of these individuals conducted additional tests, including 11 biopsies. Six of these biopsies were normal; however, three were pre-cancerous and two were malignant (basal cell carcinoma). Therefore, five participants who pursued additional clinical care as a result of their CPMC melanoma risk reports may have benefitted from earlier detection. Based on these numbers, we also have no reason to suspect any over-utilization of health care resources as a result of the CPMC melanoma risk report.

This study is not without limitations. Results may not generalize to the US population at large. In particular, self-selection bias may be introduced since participants that have chosen to join the CPMC study may be more interested in knowing their genetic risk for reported diseases, less concerned about increased genetic risk for a given disease, and more motivated to mitigate disease risk compared to the general population. The study relies on self-reported data and is, therefore, also subject to reporting bias. Due to the need for outcome survey completion, preventive behavior for those who did not view the available risk report and those who did not complete an outcome survey could not be analyzed. In addition, we did not address risk report comprehension or retention in the current study. Outcome survey completion was 36% despite a fifteen to twenty minute survey completion time. Participant recruitment, risk report viewing, and outcome survey completion occurs on a rolling basis, and we were therefore unable to control the seasonality associated with our data collection.

Results were reported for just one melanoma genetic risk variant among many possible risk alleles. Since the CPMC did not initially capture other important risk factors for melanoma, such as ultraviolet light exposure due to sun, lamps or beds, immune suppression status, presence of moles, fair skin, freckling and light hair, participants did not receive risk estimates for these factors. Moreover, the family history component of the risk report only differentiates between participants with no reported family history and participants with one or more family members with one or multiple melanomas. The outcome survey also did not capture the magnitude of change in preventive behaviors.

The clinical validity and utility of genomic risk assessment in the presence of family history must be further analyzed with respect to cost and clinical effectiveness to further validate or refute the potential benefits of testing reported in this study. In addition, further research is needed to evaluate what if any additional training and educational resources will be required to support health care providers that incorporate genetic risk testing in their clinical care. Finally, it will be critical to identify interventions that achieve sustainable preventive behaviors to reduce the rising prevalence of melanoma cancer.

## 3. Experimental Section

### 3.1. Study Population

The CPMC is a prospective research study that examines the impact disease risk assessment has on behavior and ultimately on health outcomes [12,13]. CPMC participants must be at least 18 years old, have a valid email address, provide informed consent, and provide a saliva sample for genomic analysis. Participants in the current study were recruited through one of three cohorts: CPMC community cohort, OSU community cohort, or the United States Air Force Medical Service cohort. All participants have provided informed consent to enroll in the study. The respective Institutional Review Boards of each cohort approved the study protocol in addition to the Coriell Institute Institutional Review board. The study was conducted in accordance with the Declaration of Helsinki.

After providing a saliva sample for genetic testing, participants are asked to complete a series of online questionnaires about their medical history, family history, lifestyle, and demographics through the study’s secure web portal. Personalized risk results for melanoma based on a common genetic risk variant for melanoma (rs910873) [11], which was genotyped in Coriell’s CLIA certified laboratory using the Affymetrix 6.0 gene chip, and family history (requiring at least one first degree relative to have been diagnosed with the condition) were delivered to participants through a secure web based portal.

Relative risk (RR) summaries for melanoma were reported to CPMC participants independently for family history (RR = 2.2 for participants who reported a family history of melanoma in at least one first degree relative; RR = 1.0 to those without a family history) and for genetic risk (RR = 3.0 for those with two copies of the risk variant (TT), RR = 1.7 to participants with one copy of the risk variant (CT), and RR = 1.0 to subjects with 2 copies of the non-risk variant (CC)).

### 3.2. CPMC Survey

The melanoma risk report was first released to participants on 29 July 2009, and the first melanoma outcome surveys were delivered on 15 December 2010. Subsequent participants completed the optional melanoma outcome survey at least 3 months after viewing their risk results, and we included data collected through 30 June 2014 in the current study. This optional behavioral outcome survey (also see appendix A for specific questions included in the current study) included questions related to perceived lifetime risk of developing melanoma, genetic risk and family history risk included in the melanoma risk report, behavioral changes made after viewing the melanoma risk report, motivation for making changes after viewing the melanoma risk report, and anxiety levels after viewing the melanoma risk report. For every 500 participants that completed the melanoma outcome survey, one participant was chosen to receive a $25 Amazon gift card; in total, over 1000 participants completed the melanoma outcome survey, and two gift cards were awarded. All participant recruitment, risk report delivery and viewing, and outcome survey completion was conducted on a rolling basis.

### 3.3. Exclusion Criteria

Any participants that did not have a current consent form, reported having a diagnosis of melanoma, did not view or remember viewing their reports, or did not complete the outcome survey were removed from all analyses described below. Additional exclusions include: participants who indicated they had genetic or family history risk of “don’t know” or “do not want to answer”. In total, we retained information for 718 participants in the analyses reported in the current study (see Table 1 for demographic details).

### 3.4. Data Analysis

All data analysis included in the current study was performed in 2014. We recoded the four preventive behaviors into a single yes/no binary trait reflecting whether participants increased preventive behavioral change (requiring one or more of the following: decreased sun exposure, increased use of sunscreen, increased use of protective clothing, increased frequency of skin self-exams). We also constructed a categorical reported risk variable that captured whether a participant specified that they received a risk report including one or two of the genetic risk variants (the rs910873 T allele) and/or family history risk. Since only a small fraction of participants (20/718) reported having two copies of the genetic risk variant, we combined any participant reporting at least one genetic risk variant into a single genetic risk category resulting in a total of four risk categories: no risk, genetic only risk, family history only risk, both genetic and family history risk.

We used binomial logistic regression as implemented in the glm function in R [19] to evaluate the contributions of anxiety and reported risk after correcting for demographic covariates (age, gender, and recruitment cohort). To generate all pairwise comparisons among the four reported risk categories we ran a smaller model (behavioral_change ~ reported risk) using glm in combination with the glht function in the multcomp R library [20].

The mediation model was implemented with an SPSS macro procedure, PROCESS [15,21,22] with the logistic regression function.

## 4. Conclusions

In conclusion, we have used self-reported data collected through the CPMC to explore the relationship between genetic and family history risk factors for melanoma and preventive behavior change. We found that there is a significant association that is partially mediated by anxiety, and participants were more likely to report being motivated by their genetic risk for melanoma. Our results suggest that genetic testing for melanoma risk variants complement the existing recommended utilization of family history risk, and may be even more important in cases where family history is not known completely or correctly.

## Appendix

**Table A1:** Logistic regression modeling results for sun protection and melanoma risk.

	OR	eta	SE	z Value	*p* Value
(Intercept)	0.18	−1.70	0.37	−4.58	4.55 e-06
family history (*vs.* no risk)	2.04	0.71	0.30	2.35	0.02
genetic (*vs.* no risk)	1.90	0.64	0.24	2.65	7.99 e-03
both (*vs.* no risk)	3.44	1.24	0.33	3.78	1.57 e-04

**Table A2:** Logistic regression modeling results for skin self-exams and melanoma risk.

	OR	eta	SE	z Value	*p* Value
(Intercept)	0.12	−2.14	0.43	−5.00	5.66 e-07
family history (*vs.* no risk)	2.10	0.74	0.33	2.26	0.02
genetic (*vs.* no risk)	3.43	1.23	0.25	4.90	9.41 e-07
both (*vs.* no risk)	5.23	1.67	0.32	5.14	2.78 e-07

On a scale of 1 to 5, where 1 is certain NOT to happen and 5 is certain TO happen, what do you think is your chance of developing melanoma in your lifetime?

1
2
3
4
5
Do not want to answer
Did your CPMC result show that you have a genetic risk variant for melanoma?
Yes, 1 copy
Yes, 2 copies
No
Do not know
Do not want to answer

Did your CPMC result show that you have an increased risk for melanoma based on your family history?
Yes
No
Do not know
Do not want to answer

Have you shared your CPMC result for melanoma with a health care provider?
Yes
No
Not yet, but I am planning to
Do not want to answer

Please answer the following questions about your lifestyle since viewing your CPMC results for melanoma. Did you make any lifestyle changes after viewing your CPMC result for melanoma?

**Table:**

	Increased	Did not Change	Decreased	Do not Want to Answer
My exposure to the sun				
My use of sunscreen				
The amount of protective clothing I wear				
The number of skin self-exams I perform				

What motivated you to change your level of exposure to the sun?
My CPMC genetic variant result for melanoma
My CPMC family history result for melanoma
I had symptoms of melanoma
My CPMC results for other conditions
My health care provider's recommendations
I have/had another type of skin cancer (basal cell, squamous cell, *etc*.)
Do not want to answer
Other

What motivated you to change your use of sunscreen?
My CPMC genetic variant result for melanoma
My CPMC family history result for melanoma
I had symptoms of melanoma
My CPMC results for other conditions
My health care provider’s recommendations
I have/had another type of skin cancer (basal cell, squamous cell, *etc*.)
Do not want to answer
Other

What motivated you to change the amount of protective clothing you wear?
My CPMC genetic variant result for melanoma
My CPMC family history result for melanoma
I had symptoms of melanoma
My CPMC results for other conditions
My health care provider’s recommendations
I have/had another type of skin cancer (basal cell, squamous cell, *etc*.)
Do not want to answer
Other

What motivated you to change the number of skin self exams you perform?
My CPMC genetic variant result for melanoma
My CPMC family history result for melanoma
I had symptoms of melanoma
My CPMC results for other conditions
My health care provider’s recommendations
I have/had another type of skin cancer (basal cell, squamous cell, *etc.*)
Do not want to answer
Other

Please indicate on the following scale the level of anxiety, if any, you felt immediately after viewing your CPMC result report for melanoma:
None
Low
Moderate
High
Very High
Do not want to answer
